# Examining the effects of student-centered flipped classroom in physiology education

**DOI:** 10.1186/s12909-023-04166-8

**Published:** 2023-04-12

**Authors:** Chunmei Lu, Jie Xu, Yang Cao, Ying Zhang, Xiaoyu Liu, Haixia Wen, Yan Yan, Jiao Wang, Minghui Cai, Hui Zhu

**Affiliations:** Department of Physiology, School of Basic Medical Sciences, Harbin Medical University, 157 Baojian Rd, Nangang, Harbin, Heilongjiang 150081 China

**Keywords:** Flipped classroom, Online teaching, Physiology education, Teaching model

## Abstract

**Background:**

The flipped classroom approach has gained increasing popularity in medical education. Physiology is a basic medical course that studies the phenomena and laws of human life activities, and is a crucial link course connecting preclinical courses and clinical courses. However, there is a paucity of data showing the effectiveness of the flipped classroom model for the entirety of physiology course in medical undergraduate students.

**Method:**

131 sophomore students with clinical medicine major at Harbin Medical University were recruited and they were randomly allocated into two groups: the control group which was subjected to traditional lecture teaching (n = 69), and the experimental group which was subjected to flipped classroom teaching (n = 62). To assess the effect of flipped teaching, the usual performance and final exam scores were used to evaluate the physiology learning effectiveness of students. The correlation between the usual performance and final exam scores by Pearson method was also conducted in the two teaching groups. After course completion, an anonymous questionnaire survey was conducted among the subjects of flipped classroom group to assess students’ opinion regarding the flipped classroom teaching.

**Results:**

Our results showed that the usual performance and final exam scores of students in the flipped classroom were both significantly higher than that in the traditional teaching class (*P* < 0.05). Moreover, our results also showed that the usual performance of students was significantly correlated with the final exam scores in the flipped classroom (r = 0.3945, *P* < 0.01), but not in the traditional teaching group (r = 0.1522, *P* = 0.2119). The results of questionnaire survey showed that 77.58% of the students believed flipped classroom teaching improved their knowledge acquisition. 70%~86% of students perceived that flipped classroom enhanced their learning abilities, including self-study ability, collaborative learning and problem-solving skills, and clinical thinking ability. In addition, about 60% of students acknowledged the teaching design and teaching environment, more students’ engagement and presentation of group learning in the flipped classroom.

**Conclusion:**

The flipped classroom teaching significantly improved students’ learning effectiveness in physiology course, as indicated by final exam score and usual performance. It also promoted higher-order ability-set acquisition and allowed a rationalized formative evaluation system.

## Introduction

Physiology is one of basic medical sciences that studies the phenomenon and laws of human life activities, which provides a basic understanding of how the human body functions under conditions of health and disease [1]. Physiology plays an important role as a link between preclinical courses and subsequent clinical courses. So, the understanding and mastery of physiological knowledge can affect the study of subsequent medical courses [2]. However, the curriculum content of physiology is characterized by abstract, complex, and strong logic [1, 3], and the traditional physiology teaching model has limited students’ knowledge and skills gain, because in the conventional physiology education, students passively accept knowledge during classroom time by teacher-centered lecture, so that typically without independent thinking and learning. Besides, there is no sufficient time in conventional classroom for discussion and interaction with their peers and teachers so that untimely solving student’s questions. Moreover, the traditional teaching assessment is primarily indicated by the final exam scores, which is unable to make a comprehensive and accurate evaluation over the whole learning process. Therefore, it is necessary to apply innovative instructional strategies to improve student’s learning effectiveness in this challenging course.

In recent years, the flipped classroom method to teaching has received increasingly attention in health profession education. Flipped classroom is a new educational approach invented under the background of “internet+”, which is referred to a “student-centered” pedagogical model in which classroom and self-study time are reversed [4]. Compared with teacher-centered didactic lectures in the traditional classroom model, the flipped classroom gives students access to the learning material prior to formal class time and in-class time is focused on application, stimulation, case-based discussion and problem solving [4, 5]. In the conventional teaching method where students first attend a teacher-centered lecture and the learning then be reinforced by homework that usually has no interaction with classmates or teachers [6]. In the flipped classroom model, educational content is offloaded for students to learn on their own before formal class by videos, readings, or PowerPoint slides. The subsequent face-to-face classroom time is then devoted to engaging students in interactive activities that allow students to apply their newly gained knowledge to challenging problems with their peers, so that it allows for active learning, cooperative learning, collaborative learning, and problem-based learning during classroom time [5].

As the benefits of flipped classroom includes better self-regulated learning skills, more effective student-teacher interactions, and increased learner engagement and motivation [7, 8], its use is increasingly being adopted in medical education, such as nursing [9], pharmacology [10], ophthalmology [11, 12], radiology, epidemiology, and podiatric medical education [13, 14]. To date, only several studies have reported implementation of flipped classroom in the physiology context among different level of learners [15–18], with focus on the study of system physiology. For example, Johnathan et al. has assessed the effectiveness of a modified flipped classroom curriculum cardiovascular, respiratory, and renal physiology in graduate students [18]. However, the application of the flipped classroom for the entirety of physiology course in medical undergraduate students has not been well investigated. Furthermore, no prior studies have examined the impact of flipped classroom model on the formative teaching assessment.

Consequently, in this study we report and describe the implementation and delivery of a student-centered flipped classroom methodology for the whole of physiology course in medical undergraduate students. The purpose of current study was: (1) to investigate the effectiveness of flipped classroom on physiology knowledge and skill-set acquisition as measured by usual performance and final exam score, and to determine the effect of flipped classroom model on the formative assessment system; (2) to assess the student’s perceptions of the flipped approach after course completion.

## Methods

### Subjects

We conducted this study among students with clinical medicine major in 2020 at Harbin Medical University. There are 460 students enrolled in clinical medicine major in 2020 and they are organized into 23 teaching classes by the Office of Medical Educational Administration with around 20 students in each class. We randomly selected 6 teaching classes with a total of 131 students. These participants were randomly allocated into two groups, one of which was subjected to traditional lecture teaching (TL), and the other one was subjected to flipped classroom teaching (FC). They have all finished the same pre-enrollment education and admitted to Harbin Medical University through an entrance examination, and started the physiology course in the third semester. All students were unaware of their group assignments before the course.

### Study design

131 students majoring in clinical medicine at Harbin Medical University were enrolled in this study. They are randomly allocated into either the flipped classroom group (n = 62) or the traditional lecture-based classroom group (n = 69). To assess the effect of flipped teaching, the usual performance and final exam results were used to evaluate the physiology learning effectiveness of students in these two groups. At the same time, the correlation between the usual performance and final exam scores was also determined in flipped classroom group. An anonymous questionnaire survey among the subjects of flipped classroom group was conducted to assess students’ opinion regarding the flipped teaching after course completion.

### Interventions

The two groups were both taught by the same experienced teachers using the same teaching syllabus, weekly calendar, and course content. The entirety of physiology teaching adopted flipped classroom teaching in the experimental group. For communication between students and teachers, we established a Chaoxing Group (i.chaoxing.com) in flipped classroom teaching students. The task list and learning materials for self-directed learning were offloaded for students prior to face-to-face classroom through the Chaoxing Group.

The major learning materials for flipped classroom teaching is the physiology online resource created by ourselves (https://www.xueyinonline.com/detail/232545944), which comes from “Xueyin Online”, a public MOOC platform operated by Beijing Xueyin Online Education Technology Co., Ltd (https://www.xueyinonline.com/). Our physiology course resources include 85 recorded micro-videos, 9 virtual simulation experiment teaching videos, 12 courseware and 12 sets of chapter test questions. These reading materials and videos cover all the teaching content of physiology and students can learn at their own pace. The flipped classroom students were required to follow the self-study task list by readings, watching the video, or completing the exercises to gain the basic knowledge before formal classroom time. In the face-to-face classroom, students are engaged in challenging problems using the newly gained knowledge by group discussion with their peers and the feedback and guidance from the teachers. So that students were divided into small groups (5–6 people in each group) for discussion and a representative was selected from each group to summarize and report the results of group discussion. The teachers gave comments and in-depth explanation on the discussion and answers of the students. After classroom, students are required to complete assignments and open-ended questions to reinforce and expand knowledge. The traditional teaching group, on the other hand, was mainly subjected to a teacher-centered lectures without pre-class learning and in-class group discussion. The traditional group students also had a Chaoxing Group and had access to our physiology course resources. The final exam paper was prepared by experienced teachers who did not participate in this study. The types of questions include single-choice questions, multiple-choices questions, short answer questions, and essay questions, with a total of 100 points, accounting for 60% of the final grades. The usual performance accounts for another 40% of final grades, which includes performances in video watching, classroom discussion, homework, quizzes, and attendance.

### Data collection

The usual performance as indicated by “online score”, was automatically collected by “Xueyin Online” platform, according to students’ performance in video watching (20 points), discussion (10 points), homework (20 points), quizzes (20 points), attendance (10 points) and classroom activities (20 points). For the review of final exam papers, an online test paper management platform named “Tangyun” (http://210.47.245.73/Home/Index) was used. All subjective questions including short answer questions and assay questions were reviewed by two independent blinded teachers to eliminate the subjective deviation in the teachers’ judgment. Besides, feedback from the flipped classroom students was collected using Questionnaire Star (an online tool for anonymous questionnaire surveys) [19, 20]. A total of 58 valid questionnaires were collected from the flipped classroom group with a recovery rate of 93.5%.

### Data analysis

The online scores and final exam scores were expressed in the form of mean ± standard error (SEM) and data were statistically analyzed using GraphPad Prism 8 software. An independent *t* test was used to compare mean scores between traditional teaching group and flipped classroom group. The correlation between students’ online scores and final exam scores in the two teaching groups was analyzed by Pearson method. A level of *P* < 0.05 was considered statistically significant.

## Results

### Flipped classroom improves students’ learning effectiveness in physiology course

To evaluate the impact of flipped classroom on the student’s learning effectiveness, students’ online scores and final exam scores (including scores in objective questions and subjective questions) were compared between the flipped classroom group and traditional teaching group. Our results showed that the average online score of students in the flipped classroom was significantly higher than that in traditional teaching class (94.82 ± 0.67 vs. 91.97 ± 1.28, *P* < 0.05, see Table 1).

There was also a significant increase in the final exam score of students from flipped classroom compared to that from traditional teaching group (85.47 ± 1.28 vs. 81.46 ± 1.27, *P* < 0.05, see Table 1). Moreover, our results showed that the average scores in objective questions of students from flipped classroom were significantly higher than that of traditional teaching group. (42.81 ± 0.61 vs. 40.72 ± 0.57, *P* < 0.01). It was worth noting that the students of flipped classroom also had higher subjective question scores than that of traditional teaching group. (42.67 ± 0.75 vs. 40.74 ± 0.78, *P* < 0.05).

**Table 1:** The average scores of students in the traditional teaching group and flipped classroom teaching group

Group	Online score	Objective score	Subjective score	Final exam score
Traditional teaching (n = 69)	91.97 ± 1.28	40.72 ± 0.57	40.74 ± 0.78	81.46 ± 1.27
Flipped classroom (n = 62)	94.82 ± 0.67*	42.81 ± 0.61**	42.67 ± 0.75*	85.47 ± 1.28*

Values are means ± SEM and analyzed by t-test. *P < 0.05, ** *P* < 0.01, flipped classroom vs. traditional teaching.

We further analyzed the distribution of students with different final exam scores in the two teaching classes. A score of 90 ~ 100 points is excellent, 80 ~ 89 points is good, 70 ~ 79 points is average, 60 ~ 69 points is fair, and lower than 60 points is failure. As shown in Table 2, the percentage of excellent in flipped classroom (30, 48%) was higher than that in traditional teaching class (19, 28%). Correspondingly, the student’s number of failure and fair in the flipped classroom was lower than that in traditional teaching class (6, 9.7% vs. 11, 15.9%).

**Table 2:** Number of students with different level of final exam score in traditional teaching group and flipped classroom group

Group	Excellent (90 ~ 100)	Good (80 ~ 89)	Average (70 ~ 79)	Fair (60 ~ 69)	Failure (< 60)
Traditional teaching (n = 69)	19 (28%)	23 (33.3%)	16 (23.2%)	9 (13%)	2 (2.9%)
Flipped classroom (n = 62)	30 (48%)	16 (25.8%)	10 (16.1%)	6 (9.7%)	0 (0%)

### The final exam score was correlated with online score in flipped classroom

**Fig. 1:**
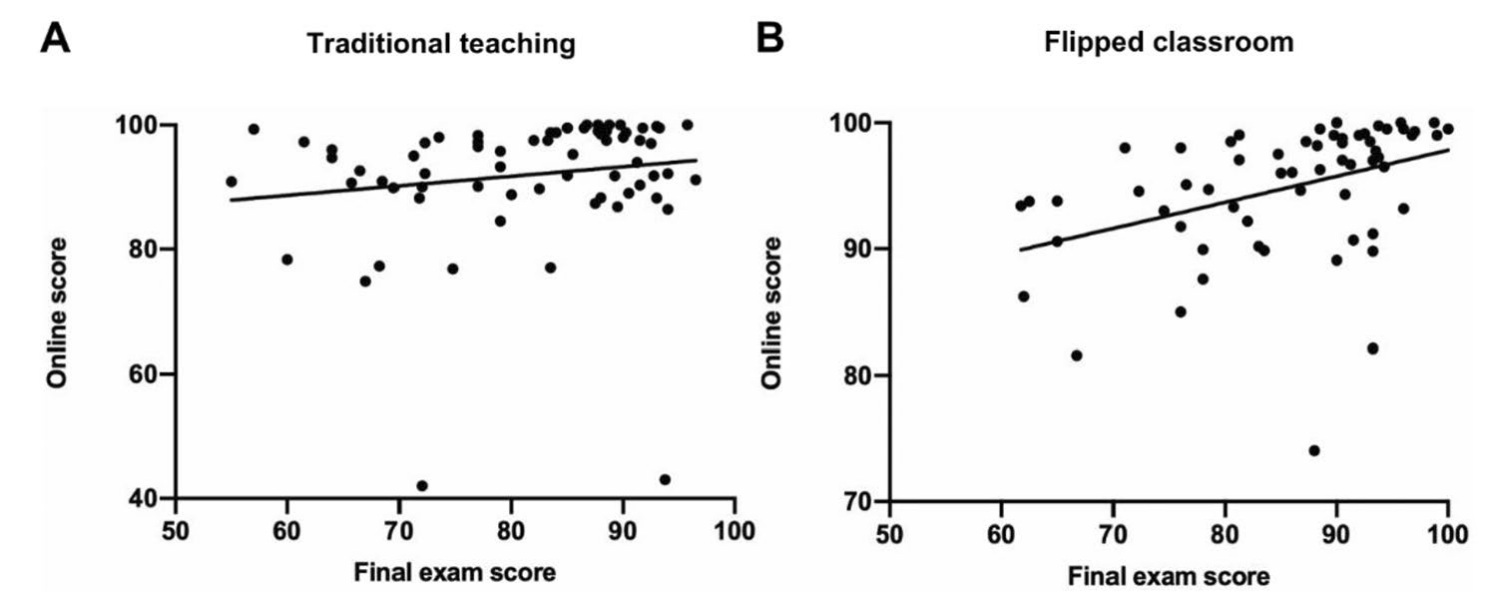
Correlation between online score and final exam score of students in the flipped classroom (n = 62) and traditional teaching class (n = 69)

In addition, we also examined the correlation between online scores and final exam scores of students by Pearson Correlation Analysis in the two teaching groups. Our results revealed that the online score of students was significantly correlated with the final exam scores in the flipped classroom (r = 0.3945, *P* < 0.01, Fig. 1B), but not in the traditional teaching class (r = 0.1522, *P* = 0.2119, Fig. 1A), suggesting flipped classroom teaching enhanced students final exam scores may be due to its positive impact on the usual performance of students.

### Students’ evaluation on flipped classroom teaching in physiology course

**Fig. 2:**
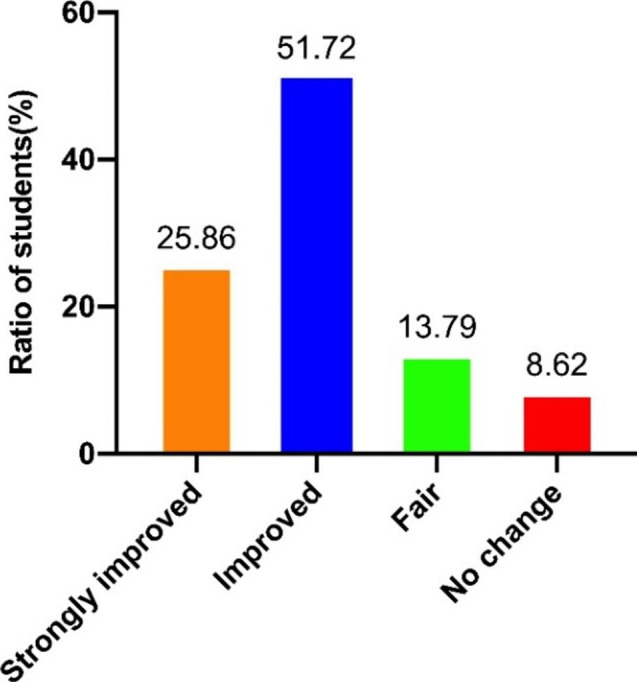
Survey of flipped classroom teaching on the improvement of students’ knowledge acquisition

All the students in the flipped classroom (n = 62) were invited to complete a questionnaire, and 58 students (93.5%) responded. First of all, the results showed that 77.58% of the students thought the flipped classroom teaching improved their knowledge acquisition. There were only 8.62% who thought that flipped classroom teaching had little impact on physiology knowledge gain (Fig. 2).

**Fig. 3:**
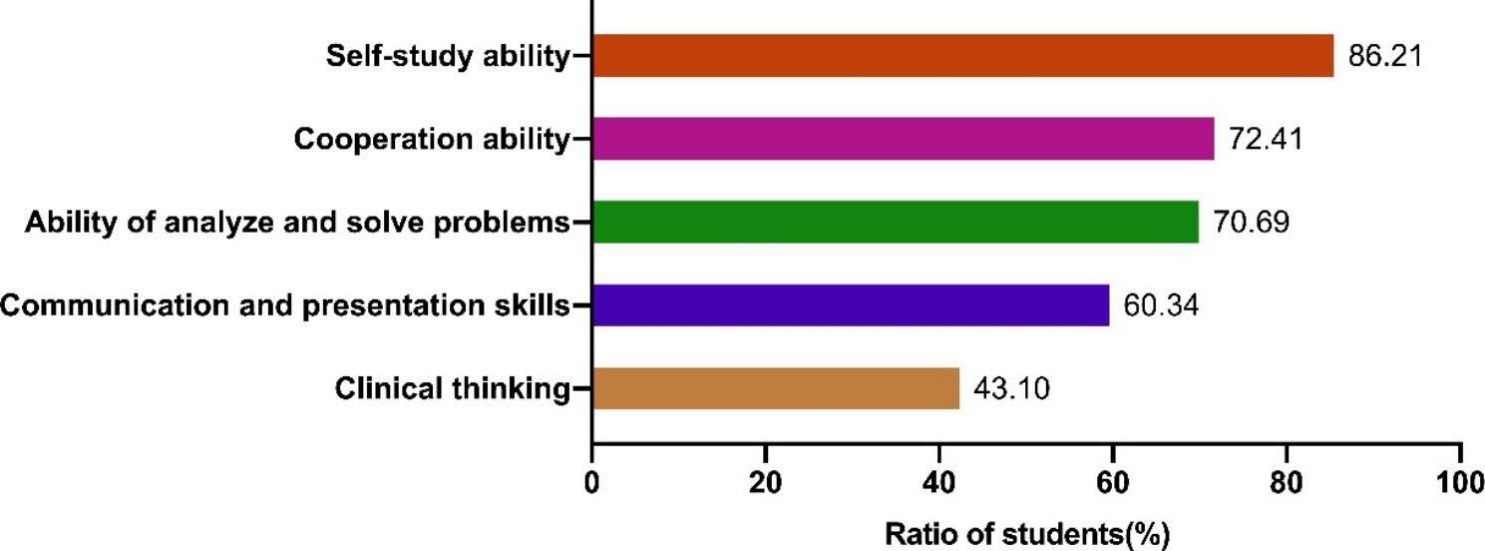
Survey of flipped classroom teaching on the students’ learning ability

Importantly, most of students perceived that flipped classroom teaching improved their learning abilities, including self-study ability (86.21%), collaborative and problem-solving skills (~ 70%), communication and presentation skills (60.34%), and clinical thinking ability (43.1%) (Fig. 3).

**Fig. 4:**
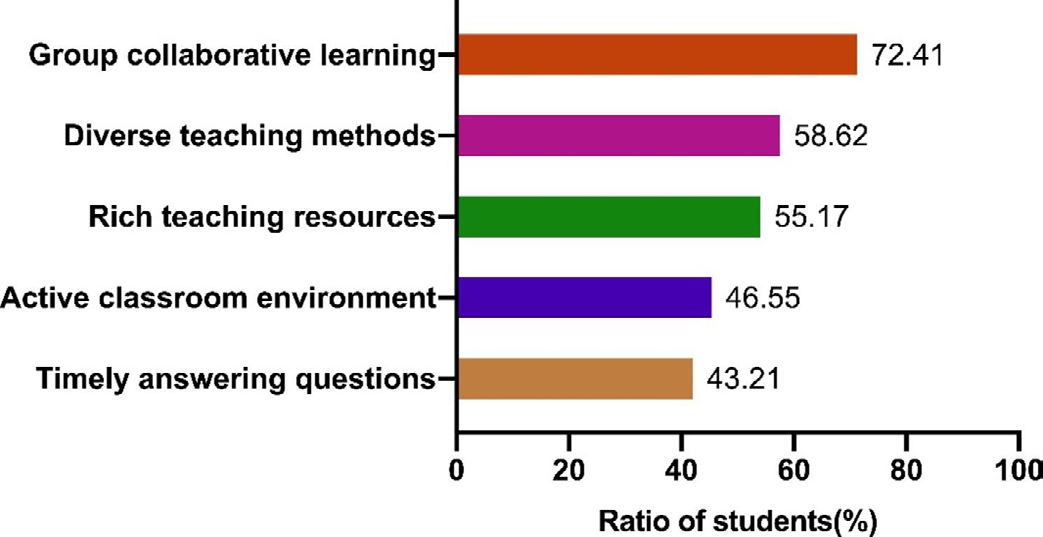
Survey on the potential contributors of flipped classroom teaching in promoting learning effectiveness in physiology

Further survey on the potential contributors of flipped classroom in enhancing learning effectiveness revealed that group collaborative learning (72.41%) was the most dominant contributor. In addition, about 50% of the students thought that flipped classroom helped to improve learning effectiveness by the diverse teaching methods (58.62%), rich teaching resources (55.17%), and active classroom environment (46.55%) (Fig. 4).

**Fig. 5:**
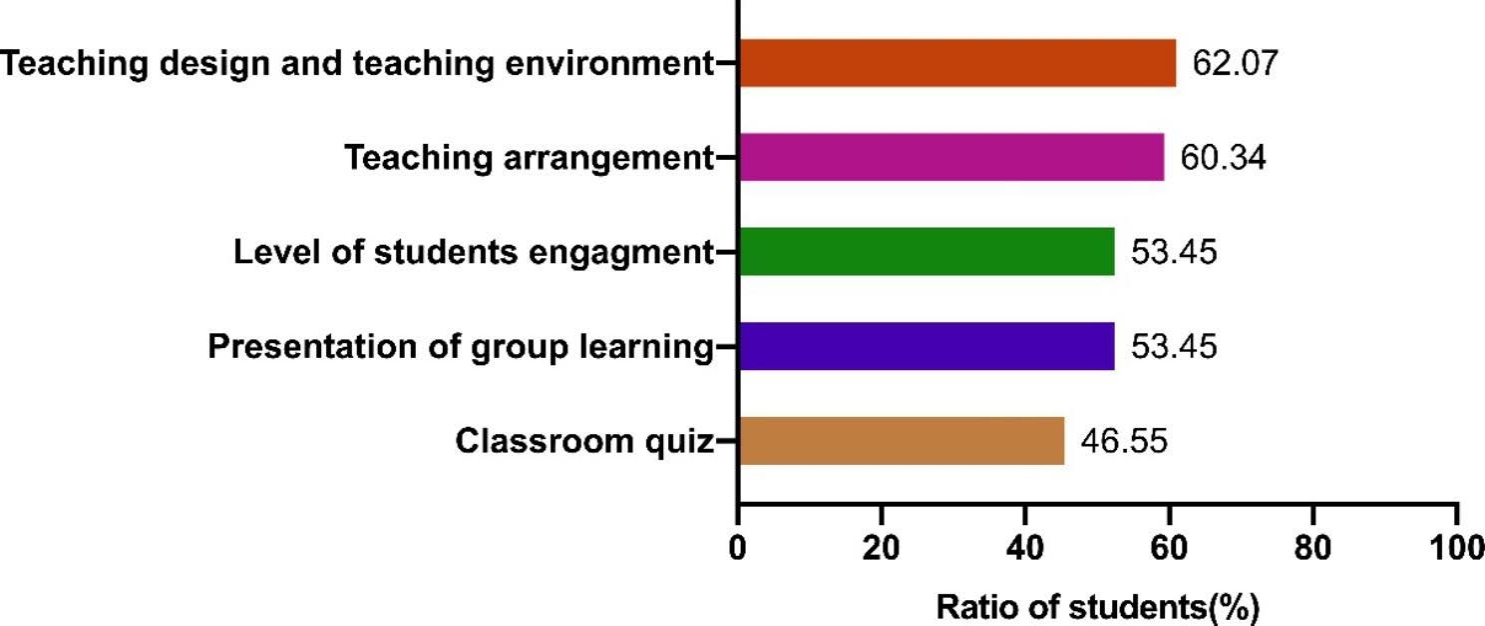
Survey of students’ evaluation on advantages of flipped classroom teaching

We also surveyed the potential characteristics of flipped classroom teaching that attract students. About 60% of student showed positive attitudes toward teaching design and teaching environment (62.07%) and teaching arrangement (60.34%). Over 50% of students acknowledged the level of students’ engagement and presentation of group learning in the flipped classroom teaching (Fig. 5).

## Discussion

Our results demonstrate that flipped classroom teaching approach is an effective and well-accepted modality for the entirety of physiology education. It not only improves the learning outcome of physiology including student’s academic performance and learning skills acquisition, but also provides better formative assessment on the whole learning process. Furthermore, most students who participated showed positive attitudes toward flipped classroom teaching in physiology course.

### Flipped classroom can improve students’ learning outcomes

Performance of the final examination in the present study suggested that the flipped classroom technique enhanced mastery of physiology content. Our results also showed that the final exam scores were correlated with students’ usual performance in the flipped classroom but not in the conventional teaching class, suggesting that the improvement of learning outcome may be due to its positive effects on students’ usual performance of flipped classroom teaching. The effectiveness of flipped classroom teaching has been increasingly investigated in medical education. Several studies proved that flipped classroom teaching was a successful approach to medical education for ophthalmology, nursing, and pharmacology etc. [9–11] Researchers found that perception of students toward medical teaching could be greatly improved in flipped classroom. It helped students achieve better academic performance in their learning [21, 22]. Recently, a study also indicated that flipped classroom teaching increased students’ learning effectiveness in physiology course, and facilitated the learning of the follow-up medical courses in long-term [2]. This may be associated with the more motivation and participation, better self-directed study and deep discussion in flipped classroom.

The effectiveness of flipped classroom teaching can also be attributed to high-quality online resources [23]. In our study, the major online learning materials is the physiology online resource created by ourselves, which including micro-video lectures, knowledge outlines, courseware and exercises etc. It allows students to click the file and learn on their own at any time and any place. Furthermore, students can rewind repeatedly to deepen and consolidate knowledge. So, our physiology online resources allow personalized learning and promote the implementation of flipped classroom. In addition, our questionnaire results suggest that the success of flipped classroom for physiology may be also associated with appropriate teaching design and arrangement, and active teaching environment in this teaching method.

### Flipped classroom is helpful to the development of students’ abilities

Medical students are required to master basic knowledge as well as learning abilities for the follow-up clinical practice [24]. Our questionnaire survey showed that most of students who participated perceived that flipped classroom teaching improved their skills-set gain, including self-learning ability, collaborative and problem-solving skill, communication and presentation skill, and clinical thinking skill.

Flipped classroom teaching method can promote the self-learning ability because students are asked to follow the self-study task list via online resources to gain the basic knowledge before class. During the face-to-face class time, students are devoted to resolving problems using the new knowledge by group discussion with their peers. The teachers gave comments and guidance on the discussion and answers of the students to expand knowledge. The interactive activities between students and teachers improve students’ higher-order abilities acquisition such as cooperative and collaborative skill, problem-solving skill, and clinical thinking ability in flipped classroom [25, 26].

### Flipped classroom allows a formative evaluation system

A scientific teaching evaluation system can effectively stimulate the engagement of learners and provide important guarantee for talent training [27]. In contrast to the traditional physiology teaching in which the assessment of students’ learning outcome is mainly indicated by their performance of final exam, the flipped classroom approach allows to establish a formative evaluation system over the whole learning process. Because the teaching process of flipped classroom is consist of pre-class, in-class and after-class. The usual performance of students before, during, and after class can be assessed by the teachers or collected by the online learning platform. In our research, the final grades of physiology course are the sum of usual performance (40%) and final exam scores (60%), which is considered as a formative evaluation method on the learning effectiveness of physiology. Compared with traditional evaluation method, this formative evaluation system can effectively encourage students’ enthusiasm for learning, help teacher to adjust their teaching plans at the time needed, and significantly improve the teaching effect. Studies have shown that formative evaluation can improve the learning outcomes of students in medical education [28, 29]. Our results also demonstrated that the online scores of students were higher in the flipped classroom, and it was significantly correlated with the final exam scores, indicating that flipped classroom teaching can improve the mastery of physiology knowledge by promoting students’ usual performance in the whole learning process.

### Study limitations

Several limitations of flipped classroom teaching need to be considered. First, flipped classroom teaching may increase the workload for the students, so that some students may be anxious and lost learning motivation due to insufficient pre-class preparation time. The educators should always monitor the learning progress of students and give appropriate guidance to help students keep learning motivation. Second, some evaluations of the flipped classroom strategy are based on group-assessment rather than individual students, so that some students who did not participate in group learning and discussion may even get same scores with their peers. Therefore, an updated evaluation system would be required to assess students’ compliance (i.e., participation, engagement, attendance) during the teaching process [30]. Lastly, the number of enrolled subjects in the present study is relatively small. It might be interesting if delivering the flipped classroom teaching to all students.

## Conclusion

Flipped classroom teaching combined with our physiology online resources provides a more effective teaching approach for physiology education. It can improve students’ academic grades as indicated by final exam score and usual performance, promote higher-order ability-set acquisition, and allow a rationalized formative evaluation system. Future studies should assess the quality control of the teaching process and evaluate students’ compliance in this teaching approach.

## Data Availability

All data generated or analyzed during this study are included in this published article.

## References

[1] Noble D (2004). Commentary: physiology is the logic of life. Jpn J Physiol.

[2] Ji M, Luo Z, Feng D, Xiang Y, Xu J (2022). Short- and long-term influences of flipped Classroom Teaching in Physiology Course on Medical Students’ learning effectiveness. Front Public Health.

[3] Sinnayah P, Salcedo A, Rekhari S (2021). Reimagining physiology education with interactive content developed in H5P. Adv Physiol Educ.

[4] Chen F, Lui AM (2017). Martinelli. A systematic review of the effectiveness of flipped classrooms in medical education. Med Educ.

[5] McLaughlin JE, Roth MT, Glatt DM, Gharkholonarehe N, Davidson CA, Griffin LM, Esserman DA, Mumper RJ (2014). The flipped classroom: a course redesign to foster learning and engagement in a health professions school. Acad Med.

[6] Mazur E, Education, Farewell (2009). lecture? Sci.

[7] Oudbier J, Spaai G, Timmermans K, Boerboom T (2022). Enhancing the effectiveness of flipped classroom in health science education: a state-of-the-art review. BMC Med Educ.

[8] Zheng B, Zhang Y (2020). Self-regulated learning: the effect on medical student learning outcomes in a flipped classroom environment. BMC Med Educ.

[9] Della Ratta CB (2015). Flipping the classroom with team-based learning in undergraduate nursing education. Nurse Educ.

[10] McLaughlin JE, Griffin LM, Esserman DA, Davidson CA, Glatt DM, Roth MT, Gharkholonarehe N, Mumper RJ (2013). Pharmacy student engagement, performance, and perception in a flipped satellite classroom. Am J Pharm Educ.

[11] Alabiad CR, Moore KJ, Green DP, Kofoed M, Mechaber AJ. and C.L. Karp. The Flipped Classroom: An Innovative Approach to Medical Education in Ophthalmology. J Acad Ophthalmol (2017). 2020; 12:e96-e103. 10.1055/s-0040-1713681 PMC7869843 33564741

[12] Tang F, Chen C, Zhu Y, Zuo C, Zhong Y, Wang N, Zhou L, Zou Y, Liang D (2017). Comparison between flipped classroom and lecture-based classroom in ophthalmology clerkship. Med Educ Online.

[13] Hew KF, Lo CK (2018). Flipped classroom improves student learning in health professions education: a meta-analysis. BMC Med Educ.

[14] Marshall AM, Conroy ZE (2022). Effective and time-efficient implementation of a Flipped-Classroom in Preclinical Medical Education. Med Sci Educ.

[15] Bingen HM, Steindal SA, Krumsvik RJ, Tveit B (2020). Studying physiology within a flipped classroom: the importance of on-campus activities for nursing students’ experiences of mastery. J Clin Nurs.

[16] Joseph MA, Roach EJ, Natarajan J, Karkada S, Cayaban ARR (2021). Flipped classroom improves omani nursing students performance and satisfaction in anatomy and physiology. BMC Nurs.

[17] Xiao N, Thor D, Zheng M, Baek J, Kim G (2018). Flipped classroom narrows the performance gap between low- and high-performing dental students in physiology. Adv Physiol Educ.

[18] Tune JD, Sturek M, Basile DP (2013). Flipped classroom model improves graduate student performance in cardiovascular, respiratory, and renal physiology. Adv Physiol Educ.

[19] Huang L, Lei W, Xu F, Liu H, Yu L (2020). Emotional responses and coping strategies in nurses and nursing students during Covid-19 outbreak: a comparative study. PLoS ONE.

[20] Lin H, Li Z, Yan M (2022). Burn-out, emotional labour and psychological resilience among gastroenterology nurses during COVID-19: a cross-sectional study. BMJ Open.

[21] Bhavsar MH, Javia HN (2022). Mehta. Flipped Classroom versus Traditional Didactic Classroom in Medical Teaching: a comparative study. Cureus.

[22] Sajjad S, Gowani A (2021). Introducing a flipped classroom in a pharmacology course. Br J Nurs.

[23] Mortensen CJ, Nicholson AM (2015). The flipped classroom stimulates greater learning and is a modern 21st century approach to teaching today’s undergraduates. J Anim Sci.

[24] Angadi NB, Kavi A, Shetty K, Hashilkar NK (2019). Effectiveness of flipped classroom as a teaching-learning method among undergraduate medical students - an interventional study. J Educ Health Promot.

[25] Freeman S, Eddy SL, McDonough M, Smith MK, Okoroafor N, Jordt H (2014). Wenderoth. Active learning increases student performance in science, engineering, and mathematics. Proc Natl Acad Sci U S A.

[26] Xie F, Derakhshan A (2021). A conceptual review of positive teacher Interpersonal Communication Behaviors in the Instructional Context. Front Psychol.

[27] Watling CJ, Lingard L (2012). Toward meaningful evaluation of medical trainees: the influence of participants’ perceptions of the process. Adv Health Sci Educ Theory Pract.

[28] Abu-Zaid A (2013). Formative assessments in medical education: a medical graduate’s perspective. Perspect Med Educ.

[29] McNulty JA, Espiritu BR, Hoyt AE, Ensminger DC, Chandrasekhar AJ (2015). Associations between formative practice quizzes and summative examination outcomes in a medical anatomy course. Anat Sci Educ.

[30] Steinel NC, Corliss S, Lee MW (2022). Voluntary participation in flipped classroom application sessions has a negligible effect on assessment outcomes in an accelerated pass-fail course. Adv Physiol Educ.

